# Supplementary material for: Interoception network in the rat brain
Source: Imaging Neurosci (Camb). 2026 Sep 23;4:IMAG.a.1334. doi: 10.1162/IMAG.a.1334 (PMC13602131; doi:10.1162/IMAG.a.1334)
Supplement: Supplementary file 1 [file IMAG.a.1334_supp.pdf]

Supplementary Materials

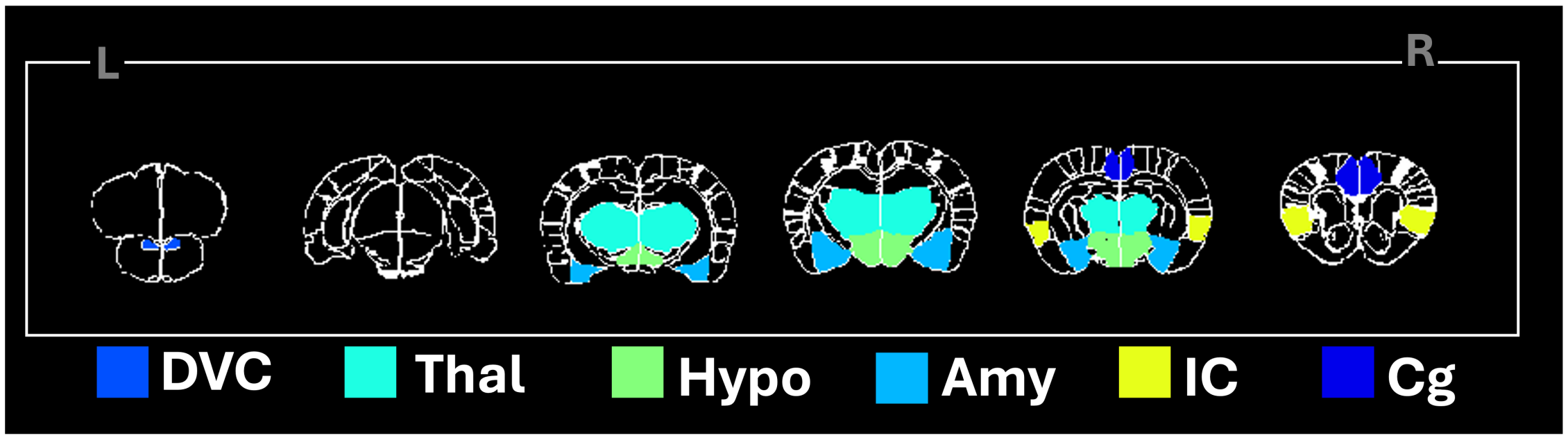

Figure S1: Anatomical ROIs are overlaid on representative brain slices: dorsal vagal complex (DVC), thalamus (Thal), hypothalamus (Hypo), amygdala (Amy), insular cortex (IC), and cingulate cortex (Cg)

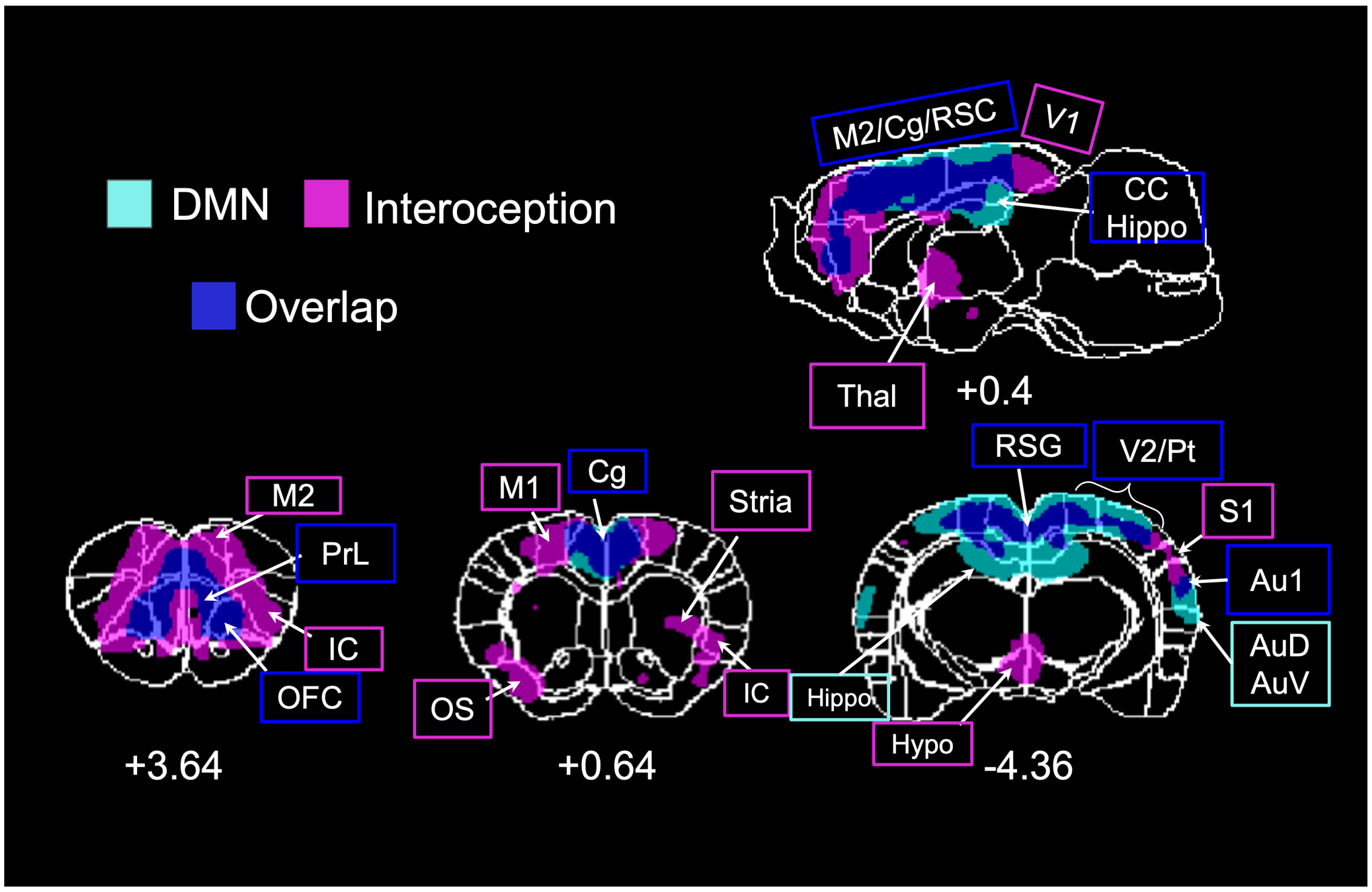

Figure S2: Partial overlap between the interoception network and the default mode network (DMN) in the rat brain. Cyan denotes regions assigned to the DMN, pink denotes regions assigned to the interoception network, and blue indicates overlapping voxels. In total, 35.5% of voxels in the interoception network are also part of the DMN. The DMN mask is derived from the DMN published in (Lu et al., 2012).

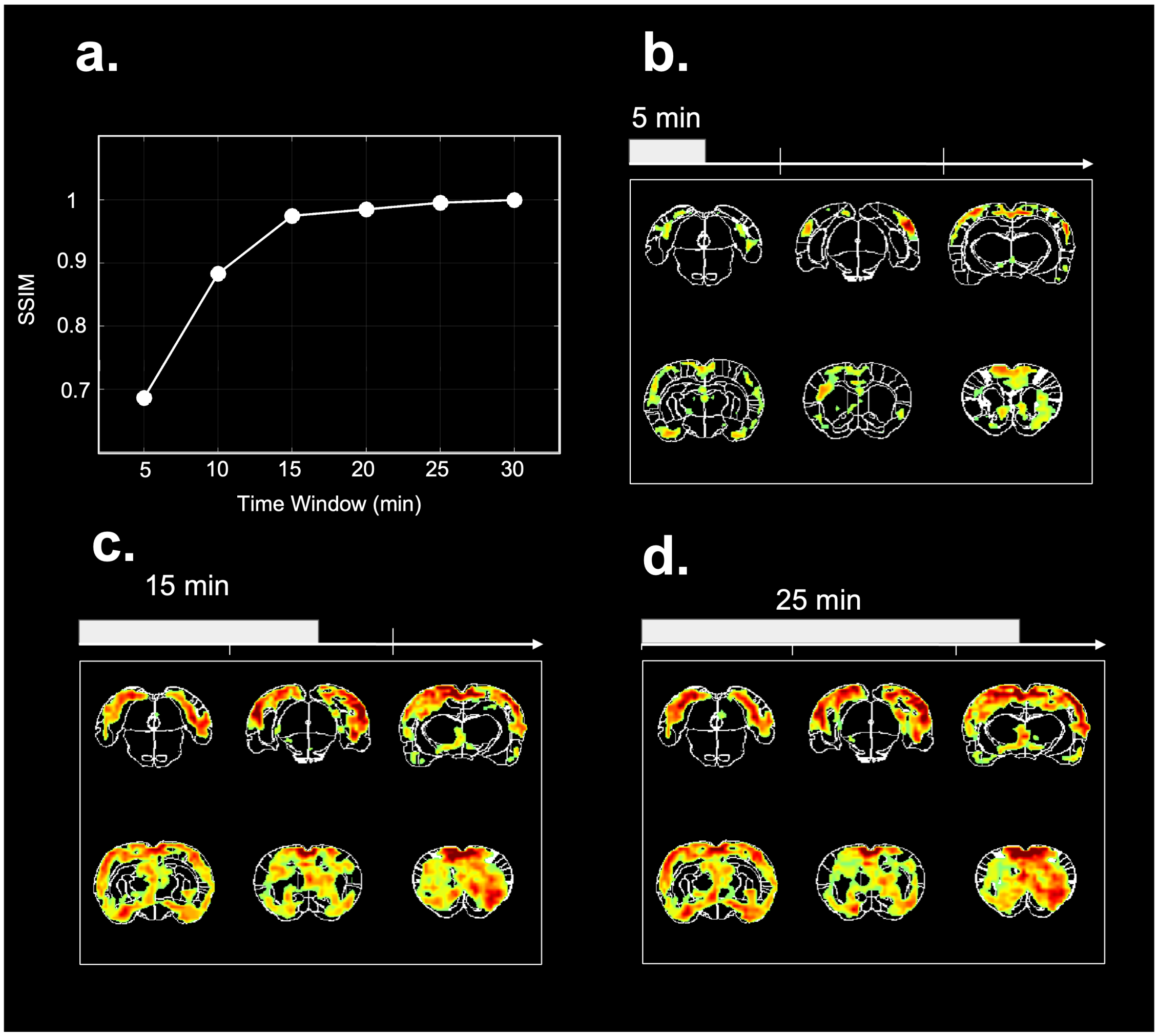

Figure S3: a.Structural Similarity Index (SSIM) at each time window relative to the 30 min window. b, c and d are conjunction maps computed with 5, 15, and 25 min windows respectively

Table S1: Brain region abbreviations and anatomical definitions used in this study.

**Abbreviation**	**Full region name**
Amy	Amygdala
Au1	Primary auditory cortex
AuV	Secondary auditory cortex, ventral area
Cg	Cingulate cortex
Cere	Cerebellum
DD	Diagonal domain
DIEnt	Dorsal intermediate entorhinal cortex
DLEnt	Dorsolateral entorhinal cortex
DVC	Dorsal vagal complex
Ect	Ectorhinal cortex
fim	Fimbria
Hippo	Hippocampus
Hypo	Hypothalamus
IC	Insular cortex
InC	Internal capsule
Iso	Isocortex
LPtA	Lateral parietal association cortex
M1	Primary motor cortex
M2	Secondary motor cortex
MPtA	Medial parietal association cortex
OFC	Orbitofrontal cortex
OS	Olfactory structures
PAG	Periaqueductal gray
PrL	Prelimbic area
PtPC	Parietal cortex, posterior area, caudal part
PtPD	Parietal cortex, posterior area, dorsal part
RSD	Retrosplenial dysgranular cortex
RSG	Retrosplenial granular cortex
S1BF	Primary somatosensory cortex, barrel field
S1DZ	Primary somatosensory cortex, dysgranular zone
S1FL	Primary somatosensory cortex, forelimb region
S1HL	Primary somatosensory cortex, hindlimb region
SN	Substantia nigra
Stria	Striatum
TeA	Temporal association cortex
Thal	Thalamus
V1	Primary visual cortex
V1B	Primary visual cortex, binocular area
V1M	Primary visual cortex, monocular area
V2L	Secondary visual cortex, lateral area

References

Lu, H., Zou, Q., Gu, H., Raichle, M. E., Stein, E. A., & Yang, Y. (2012, March). Rat brains also have a default mode network. *Proceedings of the National Academy of Sciences of the United States of America*, *109*(10), 3979–3984. doi: 10.1073/pnas.1200506109
